# Long-term trends and projections of stomach cancer burden in China: Insights from the GBD 2021 study

**DOI:** 10.1371/journal.pone.0320751

**Published:** 2025-04-08

**Authors:** Zhouwei Zhan, Bijuan Chen, Yi Zeng, Rui Huang, Jiami Yu, Zengqing Guo, Xiaoyan Lin

**Affiliations:** 1 Department of Medical Oncology, Fujian Medical University Union Hospital, Fuzhou, Fujian 350001, China; 2 Department of Medical Oncology, Clinical Oncology School of Fujian Medical University, Fujian Cancer Hospital, Fuzhou, Fujian 350014, China; 3 Department of Radiation Oncology, Clinical Oncology School of Fujian Medical University, Fujian Cancer Hospital, Fuzhou, Fujian 350014, China; 4 Department of Gastric Surgical Oncology, Clinical Oncology School of Fujian Medical University, Fujian Cancer Hospital, Fuzhou, Fujian 350014, China; 5 Digestive Endoscopy Center, Clinical Oncology School of Fujian Medical University, Fujian Cancer Hospital, Fuzhou, Fujian 350014, China,; 6 Fujian Provincial Key Laboratory of Translational Cancer Medicine, Fuzhou, Fujian 350001, China; National center for chronic and non-communicable diesease prevention and control, CHINA

## Abstract

Stomach cancer continues to be a major public health concern in China, with its incidence, prevalence, mortality, and overall burden showing notable changes over time. This study set out to analyze the long-term trends of stomach cancer from 1990 to 2021, figure out the effects of aging, epidemiological changes, and population growth, and also make projections for the future. To conduct the study, data from the Global Burden of Disease Study 2021 was used. This data allowed for the analysis of various aspects such as age-standardized incidence, prevalence, mortality rates, disability-adjusted life years (DALYs), years lived with disability (YLDs), and years of life lost (YLLs) related to stomach cancer in China. Joinpoint regression analysis was carried out to spot significant trends and turning points. Decomposition analysis was done to assess how much aging, epidemiological changes, and population growth contributed. Additionally, the Bayesian age-period-cohort (BAPC) model was employed to predict what the trends might be from 2021 to 2030. In 2021, there were 611,799 new cases of stomach cancer in China. The age-standardized incidence rate was 29.05 per 100,000 people, with males having a much higher rate of 44.48 compared to females at 15.23. The age-standardized prevalence and mortality rates were 57.22 and 21.51 per 100,000 respectively, and both were higher in males as well. There were also significant gender differences in DALYs, YLDs, and YLLs, with males shouldering a greater burden. From 1990 to 2021, the incidence and mortality rates went down, especially after 2004. Through decomposition analysis, it was found that aging led to a decrease in incidence but an increase in mortality, especially among males. Epidemiological changes caused both the incidence and mortality rates to drop, and the effect was more pronounced in males. The BAPC model forecasts that the incidence and mortality rates will continue to decline for both genders from 2021 to 2030, with a more rapid decrease in males. Overall, this study emphasizes the changing trends of the stomach cancer burden in China, the significant gender differences, and the impacts of aging, epidemiological changes, and population growth. It’s crucial to keep monitoring and implement targeted public health strategies to further reduce the burden of stomach cancer.

## Introduction

Stomach cancer, also known as gastric cancer, is one of the most common malignancies worldwide and a leading cause of cancer-related deaths. Despite global efforts to reduce its incidence, stomach cancer remains a significant public health challenge, particularly in East Asia [1]. In China, stomach cancer is the fifth most common cancer and the third leading cause of cancer mortality, accounting for a substantial burden on the healthcare system [2]. The high prevalence of Helicobacter pylori infection, dietary factors, and genetic predispositions are major contributing factors to the elevated rates of stomach cancer in this region [3–5]. Despite advances in early detection and treatment, the prognosis for stomach cancer remains poor, with a 5-year survival rate of less than 30% in advanced cases [6].

Analyzing long-term trends in stomach cancer is crucial for understanding the impact of public health interventions and guiding future strategies. The Global Burden of Disease (GBD) study provides comprehensive data on cancer incidence, prevalence, mortality, and burden, allowing for detailed analysis of temporal trends and the effects of aging, epidemiological changes, and population growth. Previous studies have shown that age-standardized incidence and mortality rates for stomach cancer have declined in many countries due to improved sanitation, widespread use of antibiotics for H. pylori, and changes in dietary habits [7]. However, the situation in China presents unique challenges and trends that require targeted analysis and intervention.

This study aims to analyze the trends in stomach cancer burden in China from 1990 to 2021, using data from the GBD 2021 [8]. We employ joinpoint regression analysis to identify significant changes and turning points in incidence, prevalence, mortality, disability-adjusted life years (DALYs), years lived with disability (YLDs), and years of life lost (YLLs) rates. Additionally, we use decomposition analysis to assess the relative contributions of aging, epidemiological changes, and population growth to these trends. Finally, we apply the Bayesian age-period-cohort (BAPC) model to predict future trends in stomach cancer incidence and mortality from 2021 to 2030. Understanding these trends and projections will help inform public health strategies and resource allocation to mitigate the burden of stomach cancer in China.

## Methods

### Data sources and study population

This study utilized data from the GBD 2021 to analyze the trends in stomach cancer burden in China from 1990 to 2021. The GBD 2021 provides comprehensive and systematically collected data on cancer incidence, prevalence, mortality, DALYs, YLDs, and YLLs across various demographics and regions [8]. The study population included all individuals in China diagnosed with stomach cancer from 1990 to 2021. Data were stratified by sex and age groups to assess specific patterns and trends within these subgroups. Age-standardized rates were calculated using the World Health Organization’s world standard population to facilitate comparisons over time and between different population groups.

### Statistical analysis

Comparative analysis was conducted between China’s trends and global trends. Joinpoint regression analysis was employed to identify significant changes and turning points in age-standardized incidence, prevalence, and mortality rates over the study period. The Joinpoint Regression Program, version 5.2.0, was used for this analysis, allowing us to detect points where a statistically significant change in the linear slope of the trend occurred [9,10]. The average annual percent change (AAPC) and corresponding 95% confidence intervals (CIs) were calculated to summarize the trends over specified intervals.

### Decomposition analysis

Decomposition analysis was conducted to evaluate the relative contributions of aging, epidemiological changes, and population growth to the observed trends in stomach cancer incidence and mortality. This method quantifies the impact of each factor by breaking down the overall change into its component parts, providing a detailed understanding of the underlying drivers of trend changes [11].

### Age-period-cohort analysis

To further understand the temporal dynamics and generational influences on stomach cancer trends, we performed an age-period-cohort (APC) analysis. This analysis helps disentangle the effects of age, time period, and birth cohort on the incidence and mortality rates. Data from the GBD study for incidence, mortality, and population estimates from 1992 to 2021 were analyzed (https://ghdx.healthdata.org/record/ihme-data/global-population-forecasts-2017-2100). GBD grouped individuals under 5 and over 95 into one category. For APC model fitting, age groups were divided into 5-year intervals (e.g., 0-4, 5-9… 95-100). We calculated total incidence, deaths, and cumulative rates for each age group over 5-year periods. APC model fitting was performed using the Epi package (version 2.51) [12] in R (version 4.3.1). Model residuals and Akaike Information Criterion (AIC) were compared to determine the optimal fit.

### Bayesian age-period-cohort model

Future projections of stomach cancer incidence and mortality from 2021 to 2030 were generated using the BAPC model. This model integrates observed data with prior distributions to produce probabilistic estimates of future trends. The projections account for age, period, and cohort effects, assuming smooth transitions between these factors over time. Priors were set for precision parameters based on a normal distribution to control variability, and the model was implemented using the Integrated Nested Laplace Approximation (INLA) method in R. Data completeness and quality were considered when selecting inputs, and sensitivity analyses were conducted to validate the robustness of the results [13,14].

### Ethical considerations

This study utilized publicly available data from the GBD 2021, and no individual-level data were accessed. Therefore, no ethical approval was required. All data handling and analysis complied with relevant guidelines and regulations.

## Results

### Incidence, prevalence, and mortality of stomach cancer in China, 2021

**Fig 1:**
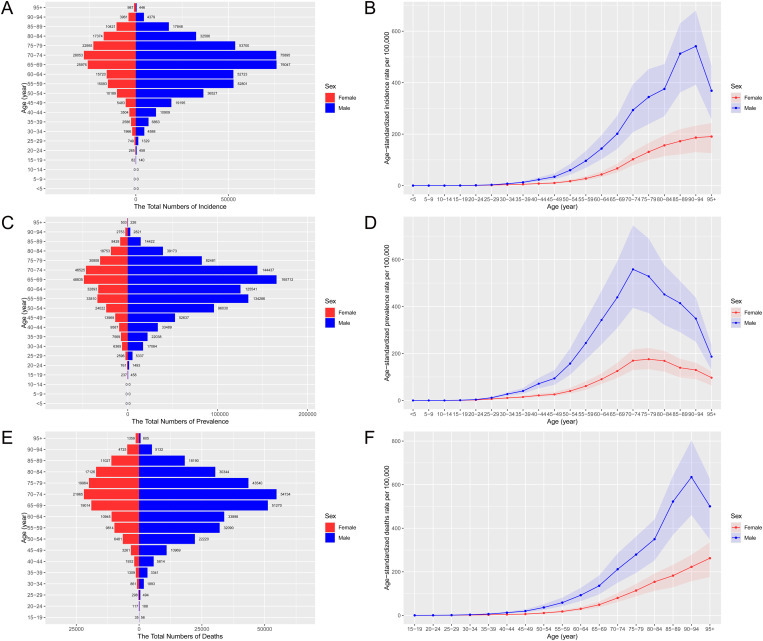
Distribution and rates of incidence, prevalence and mortality of stomach cancer in China, 2021. (A) Total new cases by age and sex. (B) age-standardized prevalence rates per 100,000 by age and sex. (C) Total prevalence cases by age and sex. (D) age-standardized prevalence rates per 100,000 by age and sex. (E) Total new deaths by age and sex. (F) age-standardized mortality rates per 100,000 by age and sex.

In 2021, stomach cancer in China exhibited significant age and sex-specific variations, presenting a substantial health burden. The total number of new stomach cancer cases was 611,799, with males accounting for 446,434 and females for 165,365. This translates to age-standardized incidence rates of 29.05 per 100,000 people overall, 44.48 for males, and 15.23 for females. Similarly, the total prevalence was 1,226,056 cases, predominantly among males (937,643), leading to age-standardized prevalence rates of 57.22 per 100,000 overall, 89.25 for males, and 26.71 for females. Mortality data revealed 445,013 deaths due to stomach cancer, with a significantly higher burden on males (314,779 deaths) compared to females (130,234 deaths), resulting in age-standardized mortality rates of 21.51 per 100,000 people overall, 32.61 for males, and 12.02 for females (Table 1). Fig 1 further illustrates these age-specific trends. Fig 1A shows the age distribution of new cases, highlighting a peak in the 70–74 age group and a higher incidence among males. age-standardized incidence rates confirmed the elevated risk for males across all age groups (Fig 1B). Similarly, Figs 1C and 1D show the prevalence patterns, with males having higher prevalence rates, particularly in older age groups. Mortality trends and age-standardized mortality rates mirrored these findings, demonstrating higher mortality rates among older males, thus emphasizing the pronounced gender disparity in the stomach cancer burden in China (Figs 1E and 1F).

**Table 1:** All-age cases and age-standardized incidence, prevalence, deaths, DALYs, YLDs and YLLs rates in 2021 for stomach cancer in China.

Measure	All-ages cases			Age-standardized rates per 100,000 people		
	Total	Male	Female	Total	Male	Female
Incidence	611799 (471966, 765562)	446434 (325932, 589284)	165365 (127716, 208140)	29.05 (22.42, 36.2)	44.48 (32.18, 58.38)	15.23 (11.77, 19.16)
Prevalence	1226056 (943897, 1546818)	937643 (683807, 1240047)	288412 (223730, 365255)	57.22 (44.18, 71.99)	89.25 (65.15, 117.49)	26.71 (20.68, 33.89)
Deaths	445013 (344736, 555834)	314779 (230725, 418722)	130234 (100509, 163561)	21.51 (16.66, 26.61)	32.61 (23.61, 42.8)	12.02 (9.29, 15.1)
DALYs	10642127 (8222106, 13383779)	7740359 (5634331, 10365104)	2901768 (2251657, 3679391)	501.26 (387.29, 627.98)	750.39 (550.9, 997.91)	268.83 (208.91, 340.98)
YLDs	162261 (108718, 224613)	120905 (75513, 171020)	41357 (26669, 58573)	7.64 (5.13, 10.57)	11.79 (7.37, 16.54)	3.82 (2.46, 5.42)
YLLs	10479865 (8094751, 13211220)	7619454 (5551448, 10208683)	2860411 (2219304, 3632563)	493.62 (381.57, 620.17)	738.6 (543.72, 982.8)	265.02 (205.67, 336.63)

DALYs, disability-adjusted life-years; YLDs, years lived with disability; YLLs, years of life lost.

### DALYs, YLDs, and YLLs of stomach cancer in China, 2021

The burden of stomach cancer in terms of DALYs, YLDs, and YLLs in 2021 also showed significant age and sex-specific differences. The total DALYs were 10,642,127, with males contributing 7,740,359 and females 2,901,768, leading to age-standardized DALYs rates of 501.26 per 100,000 overall, 750.39 for males, and 268.83 for females. YLDs were higher among males (120,905) compared to females (41,357), resulting in age-standardized YLDs rates of 7.64 per 100,000 overall, 11.79 for males, and 3.82 for females. YLLs were notably high, with 10,479,865 years lost, predominantly among males (7,619,454), reflecting age-standardized YLLs rates of 493.62 per 100,000 overall, 738.6 for males, and 265.02 for females (Table 1). S1 Fig provides a detailed view of these metrics. S1A Fig shows the age-specific distribution of DALYs, with a higher burden in older males, while S1B Fig confirms higher age-standardized DALY rates for males. S1C Fig illustrates the YLDs distribution, with males experiencing higher disability years, and S1D Fig shows age-standardized YLD rates, highlighting the greater burden on males. S1E-F Figs depict the YLLs, demonstrating significant life years lost due to stomach cancer, especially in older males, and confirming higher age-standardized YLL rates for males. These figures collectively emphasize the substantial impact of stomach cancer on both mortality and morbidity in China, with a pronounced gender disparity.

### Trends in stomach cancer burden from 1990 to 2021

**Fig 2:**
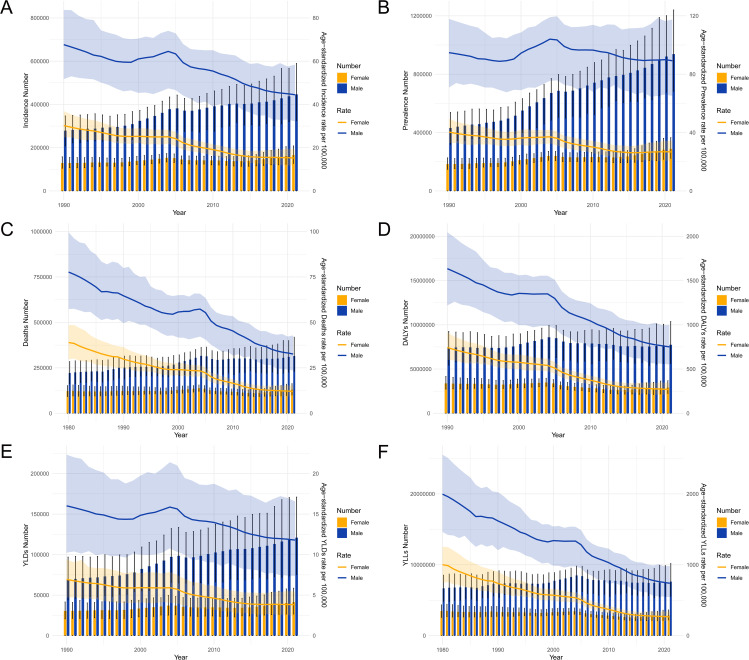
Trends in incidence, prevalence, mortality, DALYs, YLDs and YLLs of stomach cancer in China from 1990 to 2021. (A) Trends in the total number of new stomach cancer cases and age-standardized incidence rates per 100,000 by year and sex. (B) Trends in the total number of prevalent stomach cancer cases and age-standardized prevalence rates per 100,000 by year and sex. (C) Trends in the total number of deaths due to stomach cancer and age-standardized mortality rates per 100,000 by year and sex. (D) Trends in the total number of DALYs due to stomach cancer and age-standardized DALYs rate per 100,000 by year and sex. (E) Trends in the total number of YLDs due to stomach cancer and age-standardized YLDs rate per 100,000 by year and sex. (F) Trends in the total number of YLLs due to stomach cancer and age-standardized YLLs rate per 100,000 by year and sex. DALYs, disability-adjusted life years; YLDs, years lived with disability; YLLs, years of life lost.

The trends in the burden of stomach cancer in China from 1990 to 2021 show a significant increase in both the number of cases and the rates of incidence, prevalence, and mortality, particularly among males. The total number of new stomach cancer cases has risen steadily over the years, with age-standardized incidence rates per 100,000 showing higher rates for males compared to females throughout the period (Fig 2A). Prevalence trends indicate a similar pattern, with the total number of prevalent cases increasing over time and males consistently exhibiting higher age-standardized prevalence rates compared to females (Fig 2B). Mortality trends reveal a substantial rise in the number of deaths due to stomach cancer, with age-standardized mortality rates per 100,000 also showing a persistent disparity favoring higher rates in males (Fig 2C). The burden of DALYs due to stomach cancer has similarly increased, with males experiencing a greater share of the burden as evidenced by higher age-standardized DALYs rates (Fig 2D). Additionally, the number of YLDs has grown over time, with higher age-standardized YLDs rates among males (Fig 2E). The number of YLLs due to premature mortality from stomach cancer has also escalated, with males showing significantly higher age-standardized YLLs rates compared to females (Fig 2F). These findings underscore the growing public health challenge posed by stomach cancer in China, particularly the pronounced gender disparity.

### Age-specific changes in stomach cancer burden between 1990 and 2021

**Fig 3:**
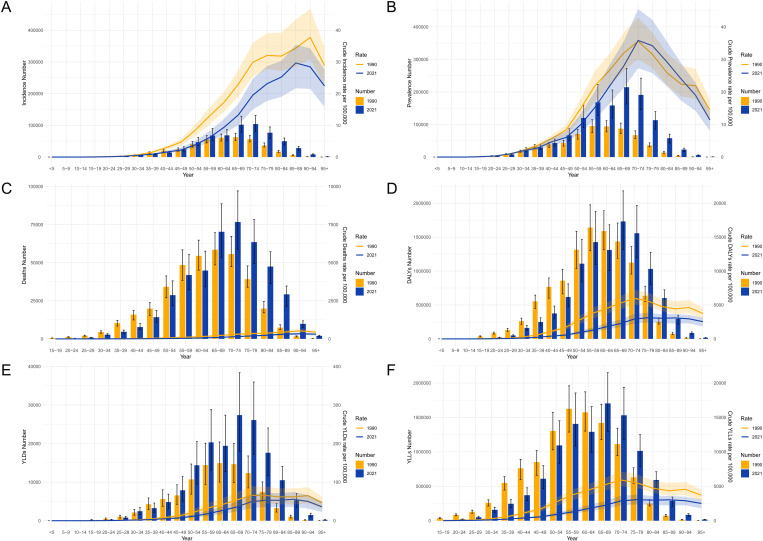
Age-specific changes in incidence, prevalence, mortality, DALYs, YLDs and YLLs of stomach cancer in China between 1990 and 2021. (A) Age-specific number of incident cases and crude incidence rates per 100,000 in 1990 and 2021. (B) Age-specific number of deaths and crude death rates per 100,000 in 1990 and 2021. (C) Age-specific number of prevalent cases and crude prevalence rates per 100,000 in 1990 and 2021. (D) Age-specific number of DALYs and crude DALYs rates per 100,000 in 1990 and 2021. (E) Age-specific number of YLDs and crude YLDs rates per 100,000 in 1990 and 2021. (F) Age-specific number of YLLs and crude YLLs rates per 100,000 in 1990 and 2021. DALYs, disability-adjusted life years; YLDs, years lived with disability; YLLs, years of life lost.

Fig 3 illustrates the age-specific changes in the burden of stomach cancer in China between 1990 and 2021, highlighting significant shifts in incidence, prevalence, mortality, and overall disease burden across different age groups. Quantitatively, incidence, prevalence, and YLDs have increased in 2021 compared to 1990 across most age groups, with minimal differences observed in individuals aged 40–44 years and younger. However, YLDs are lower in 2021 than in 1990 for those aged 40–44 years and younger (Fig 3E). In contrast, deaths, DALYs, and YLLs have decreased in 2021 compared to 1990 in individuals aged 60–64 and younger, while they have increased in the 65–69 and older age groups (Figs 3B, 3D and 3F). Crude rates for each indicator, including incidence, prevalence, mortality, DALYs, YLDs, and YLLs, are lower in 2021 than in 1990 across all age groups. These trends indicate that while the absolute burden of stomach cancer has increased, particularly among older adults, the crude rates have declined, reflecting improvements in public health interventions and cancer care over time.

### Trends in age-standardized rates of stomach cancer in China and globally

**Fig 4:**
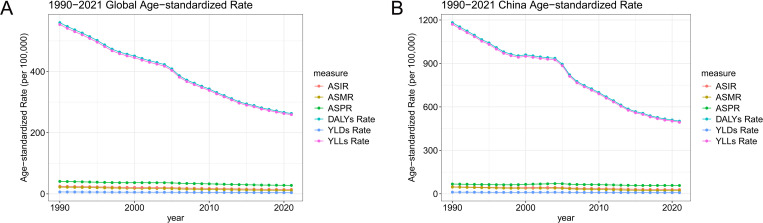
Trends in age-standardized rates of stomach cancer in China and globally from 1990 to 2021. (A) Trends in global age-standardized rates of stomach cancer incidence, prevalence, mortality, DALYs, YLDs, and YLLs per 100,000 people from 1990 to 2021. (B) Trends in China’s age-standardized rates of stomach cancer incidence, prevalence, mortality, DALYs, YLDs, and YLLs per 100,000 people from 1990 to 2021. DALYs, disability-adjusted life years; YLDs, years lived with disability; YLLs, years of life lost.

From 1990 to 2021, the age-standardized rates of stomach cancer in China and globally have shown notable changes, with several indicators reflecting significant trends (S1 Table, Fig 4). In China, the age-standardized incidence rate decreased from 48.03 to 29.05 per 100,000, while globally, it declined from 24.76 to 14.33 per 100,000, both showing significant reductions. The prevalence rate also decreased, but to a lesser extent, from 67.17 to 57.22 per 100,000 in China and from 40.64 to 27.58 per 100,000 globally. The most substantial change was observed in the age-standardized mortality rate, which fell from 46.05 to 21.51 per 100,000 in China, and from 22.01 to 11.2 per 100,000 globally, highlighting significant improvements in survival. The burden of stomach cancer measured in DALYs, YLDs, and YLLs also exhibited significant reductions. In China, the DALYs rate decreased from 1181.61 to 501.26 per 100,000, and the YLLs rate from 1170.29 to 493.62 per 100,000, both indicating substantial improvements in overall disease burden. Globally, these rates decreased from 559.72 to 262.75 per 100,000 for DALYs and from 553.58 to 258.98 per 100,000 for YLLs, reflecting similar trends. The YLDs rates also declined, though less dramatically, suggesting that while mortality has improved, the impact on long-term disability requires further attention.

### Joinpoint analysis of trends in age-standardized burden rates of stomach cancer in China from 1990 to 2021

**Fig 5:**
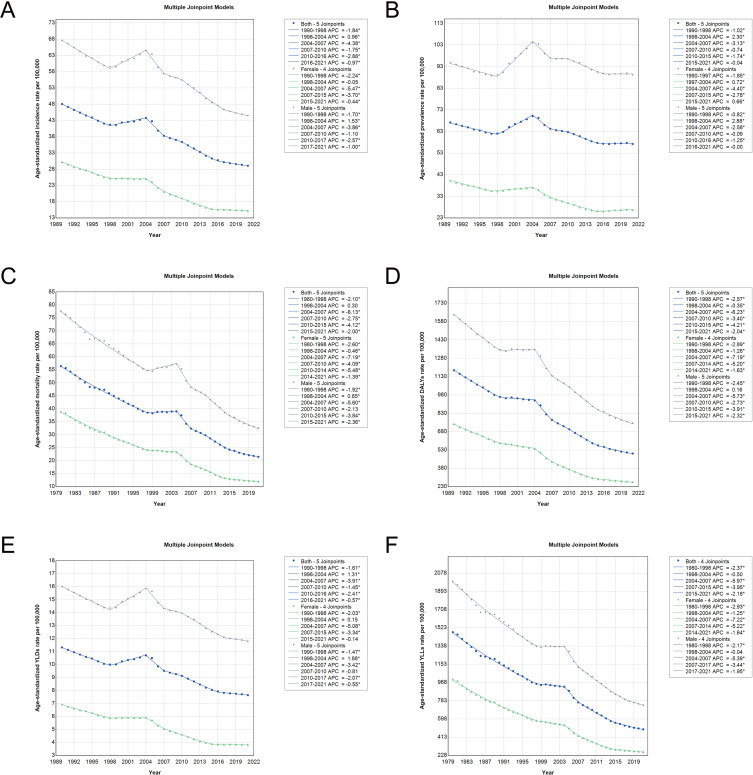
Joinpoint analysis of age-standardized incidence, prevalence, mortality, DALYs, YLDs, and YLLs rates of stomach cancer in China from 1990 to 2021 for both sexes (blue line), females (green line) and males (grey line). (A) Joinpoint analysis of age-standardized incidence rates per 100,000 from 1990 to 2021 by sex. (B) Joinpoint analysis of age-standardized prevalence rates per 100,000 from 1990 to 2021 by sex. (C) Joinpoint analysis of age-standardized mortality rates per 100,000 from 1990 to 2021 by sex. (D) Joinpoint analysis of age-standardized DALYs rates per 100,000 from 1990 to 2021 by sex. (E) Joinpoint analysis of age-standardized YLDs rates per 100,000 from 1990 to 2021 by sex. (F) Joinpoint analysis of age-standardized YLLs rates per 100,000 from 1990 to 2021 by sex. DALYs, disability-adjusted life years; YLDs, years lived with disability; YLLs, years of life lost.

The analysis of stomach cancer trends in China between 1990 and 2021 reveals notable gender differences and turning points in the progression of incidence, mortality, and other burden indicators such as DALYs, YLDs, and YLLs (S2-S3 Tables, Fig 5). Across both sexes, a general decline in age-standardized incidence and mortality rates is observed. However, females consistently show sharper decreases in these indicators compared to males. The decline in incidence and mortality appears more pronounced in females during critical periods of rapid change, particularly between 2004 and 2007, as well as between 2010 and 2015. These periods mark significant points where trends shifted substantially, reflecting potential improvements in public health interventions and cancer care. Similarly, trends in the overall disease burden, as measured by DALYs, YLDs, and YLLs, follow a downward trajectory for both genders, with females experiencing greater reductions in these indicators. The decline in YLLs, a key measure of premature mortality, is particularly notable among females, emphasizing their improved survival outcomes compared to males. This pattern is also observed in YLDs, indicating a reduction in the long-term disability associated with stomach cancer. Despite these general improvements, males continue to exhibit a higher disease burden across most indicators, underscoring ongoing gender disparities in the impact of stomach cancer.

### Age, period, and cohort effects on stomach cancer incidence and mortality

**Fig 6:**
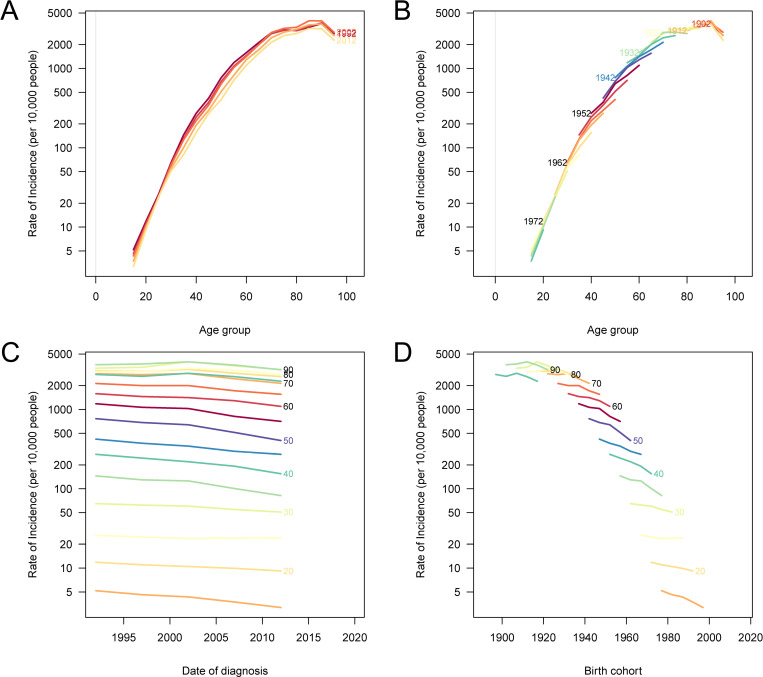
Age, period, and cohort effects on the incidence of stomach cancer in China. (A) The age-specific incidence rates of stomach cancer according to time periods; each line connects the age-specific incidence rates for a 5-year period. (B) The period-specific incidence rates of stomach cancer according to age groups; each line connects the period-specific incidence rates for a 5-year age group. (C) The cohort-specific incidence rates of stomach cancer according to age groups; each line connects the cohort-specific incidence rates for a 5-year birth cohort. (D) The age-specific incidence rates of stomach cancer according to birth cohorts; each line connects the age-specific incidence rates for a 5-year birth cohort.

The analysis of age, period, and cohort effects on stomach cancer incidence and mortality in China reveals significant variations across different age groups, birth cohorts, and time periods. Fig 6A demonstrates that the age-specific incidence rates of stomach cancer increased with age across all time periods, with the highest rates observed in older age groups. The period-specific incidence rates indicate a general decline over successive time periods for most age groups, although younger age groups showed relatively stable incidence rates (Fig 6B). The cohort-specific incidence rates highlight that more recent birth cohorts have lower incidence rates compared to earlier cohorts, particularly in older age groups (Fig 6C). Finally, Fig 6D shows that age-specific incidence rates for different birth cohorts generally increased with age, but the rates were lower for more recent cohorts. S2A Fig illustrates that age-specific mortality rates of stomach cancer also increased with age across all time periods, with the highest mortality observed in older age groups. The period-specific mortality rates reveal a decline over successive time periods, similar to the trends observed in incidence (S2B Fig). The cohort-specific mortality rates indicate that more recent birth cohorts experienced lower mortality rates, particularly in older age groups (S2C Fig). S2D Fig shows that age-specific mortality rates for different birth cohorts generally increased with age, but were lower for more recent cohorts.

### Decomposition analysis of changes in incidence and mortality of stomach cancer

The decomposition analysis of changes in stomach cancer incidence and mortality in China reveals distinct contributions from aging, epidemiological change, and population growth, with notable differences between gender groups. For incidence (S3A Fig), aging contributed to a reduction in incidence across both sexes, with a more pronounced effect in males. Epidemiological change resulted in a decrease in incidence for all groups, particularly among males. Population growth had a minor impact, slightly increasing incidence across all groups. For mortality (S3B Fig), aging played a substantial role in increasing mortality for both sexes, males, and females, with the most significant effect observed in males. Epidemiological change contributed to a decrease in mortality across all groups, with males experiencing the largest reduction. Population growth had a negligible effect on mortality. These results highlight the varying impacts of aging, epidemiological change, and population growth on the morbidity and mortality of stomach cancer between different gender groups.

### Bayesian age-period-cohort model predictions for stomach cancer

**Fig 7:**
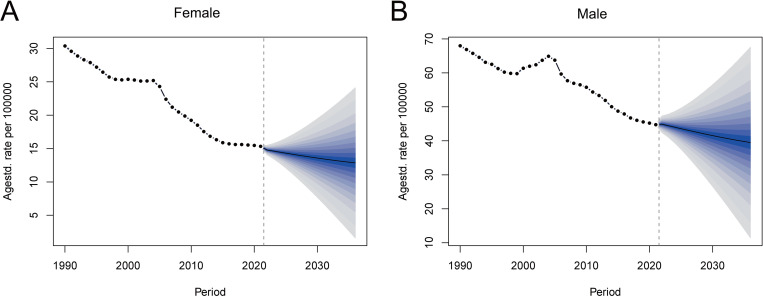
BAPC model predictions of stomach cancer incidence in China. (A) Predicted age-standardized incidence rate for females from 2021 to 2030 using the BAPC model. (B) Predicted age-standardized incidence rate for males from 2021 to 2030 using the BAPC model. BAPC, Bayesian age-period-cohort.

The BAPC model predictions for stomach cancer incidence rates in China from 2021 to 2030 reveal distinct trends for males and females. For females (Fig 7A), the model predicts a gradual decline in age-standardized incidence rates, while for males (Fig 7B), the BAPC model forecasts a more pronounced decline in age-standardized incidence rates over the same period. The projected trends indicate a continued reduction in the burden of stomach cancer for both genders, with a sharper decrease anticipated for males. These predictions highlight the dynamic changes expected in the incidence of stomach cancer in China over the next decade.

## Discussion

This study provides a comprehensive analysis of the trends in stomach cancer burden in China from 1990 to 2021, revealing significant variations in incidence, prevalence, mortality, and overall disease burden, with notable differences between genders. Our findings indicate that while the age-standardized incidence, prevalence, and mortality rates have generally declined over the study period, the absolute number of cases and deaths has increased due to population growth and aging. Males consistently exhibited higher rates across all metrics compared to females. The joinpoint regression analysis identified critical turning points in these trends, highlighting periods of significant change. Decomposition analysis revealed that aging contributed to reductions in incidence but increased mortality, particularly in males, while epidemiological changes led to decreases in both incidence and mortality. The BAPC model projections suggest a continued decline in age-standardized rates for both sexes from 2021 to 2030, with a more pronounced decrease anticipated for males. Comparisons with global trends indicate that while China has made significant progress, the burden of stomach cancer remains higher than the global average, underscoring the need for targeted public health interventions.

Our findings are consistent with previous studies that have documented a global decline in the age-standardized incidence and mortality rates of stomach cancer, primarily due to improved sanitation, reduced prevalence of Helicobacter pylori infection, and changes in dietary habits [15,16]. The trends show a decline in the population attributable fractions (PAFs) for Helicobacter pylori infection, smoking, pickled vegetable consumption, and alcohol use in relation to gastric cancer in China [17]. On the other hand, since 2005, China has gradually implemented upper gastrointestinal cancer screening. By 2019, two million individuals have benefited from standardized endoscopic screening, with 32,000 patients detected. The screening achieved early detection and treatment rates of 72.64% and 83.38%, respectively [18]. Previous studies have shown strong evidence that endoscopic screening can reduce esophageal cancer mortality by 34% and incidence by 30% [19,20].

Despite the significant decline in age-standardized rates, the burden of stomach cancer on China’s health systems has not decreased. Changes in population age structure and growth have resulted in an increase in the number of incident cases and deaths from stomach cancer in China [21]. Our study’s decomposition analysis offers additional insights by quantifying the contributions of aging, epidemiological changes, and population growth to these trends. This detailed analysis provides a deeper understanding of the drivers behind the trends observed in China, highlighting the importance of demographic factors in shaping cancer burden. Furthermore, Naghavi et al. reported that in 2017, the highest age-standardized incidence rates of stomach cancer were found in the high-income Asia Pacific and East Asia regions. Nearly half of the global incident cases occurred in China [16]. Our study emphasizes that the burden of stomach cancer in China remains above the global average, consistent with Naghavi et al.’s findings, which highlight regional variations in cancer burden.

Stomach cancer can be classified into two subsites: cardia (upper) and noncardia (lower). Chronic Helicobacter pylori infection is the main cause of noncardia gastric cancer, affecting 50% of the global population [22,23], though less than 5% develop cancer due to genetic and environmental factors [24]. Other risk factors include alcohol, smoking, and salted foods [25]. Cardia cancer, not generally associated with H. pylori, may have a dual etiology involving H. pylori and factors like excess body weight and gastroesophageal reflux disease [25]. The observed gender disparities, with males experiencing higher rates across all metrics, are well-documented in the literature [1,26]. Males have nearly twice the risk of stomach cancer compared to females, largely due to greater exposure to risk factors like smoking and alcohol consumption [27]. In China, over half of gastric cancer cases are tied to modifiable risk factors. He et al. observed declining PAFs for H. pylori infection, smoking, pickled vegetables, and alcohol consumption, but rising PAFs for unhealthy body mass index (BMI) and diabetes from 2000 to 2050. To reduce future gastric cancer burden, ongoing strategies to control these risk factors are essential [17].

We observed that China experienced a relatively small decline in age-standardized incidence rates over the study period, while the decreases in age-standardized death and attributable DALY rates were much more substantial. A study of cancer registries in China revealed that the disparity in cancer mortality rates between rural and urban areas was significantly greater than the difference in cancer incidence [28]. This disparity may result from limited medical resources, lower levels of cancer care, and a higher proportion of late-stage cancer diagnoses in rural and underdeveloped areas. China has taken steps to reduce cancer care disparities between rural and urban areas, likely explaining the sharp decrease in stomach cancer death rates [29]. Japan and South Korea have reduced stomach cancer deaths by implementing population screening programs, leading to early detection and better survival rates [30]. Some researchers suggest China should adopt similar screening strategies to reduce stomach cancer burden [31]. Our results show an increased risk of gastric cancer in adults over 40 years in China, making screening essential. However, the financial burden is high since 45.2% of China’s population is over 40. Given Helicobacter pylori’s role in gastric carcinogenesis, people over 40 can be further stratified by H. pylori infection. Financial resources could then support barium photofluorography or endoscopy screening for this high-risk group (i.e., those testing positive for H. pylori) [31].

This study has several limitations that should be acknowledged. First, it relies on data from the GBD 2021, which, while comprehensive, may have limitations in data quality and completeness, particularly for earlier years and data from rural or underrepresented regions. Second, the BAPC model and joinpoint regression analysis used in this study are based on certain assumptions, which may influence the results [14]. The accuracy of the future projections is contingent upon the validity of these assumptions, and these methodological constraints should be considered when interpreting the findings. Third, the study lacks detailed data on lifestyle factors, genetic predispositions, socioeconomic status, and the economic impact of stomach cancer, which restricts the analysis of risk factors, disparities, and the financial burden of the disease. Furthermore, this study focuses solely on China, which limits the generalizability of the findings to other regions with different epidemiological profiles, healthcare systems, and socioeconomic conditions. Comparative analyses with other countries or regions could provide valuable insights into global patterns and successful intervention strategies. Additionally, this study is unable to differentiate between cardia and non-cardia stomach cancer, which have distinct etiologies, risk factors, and implications for prevention and management [16]. Future research should address these limitations by improving the quality and comprehensiveness of cancer registries and databases, particularly in underserved regions. Incorporating genetic, environmental, and lifestyle data, as well as assessing the economic impact of stomach cancer, would provide a more holistic understanding of the disease. Finally, designing targeted public health interventions and implementing effective screening programs for high-risk populations and regions within China are critical to reducing the burden of stomach cancer and improving outcomes.

## Conclusions

This study underscores the critical importance of continued efforts to reduce the burden of stomach cancer in China, highlighting the need for targeted public health interventions. Despite significant progress, the disease remains a major public health challenge, particularly for males who experience higher rates across all metrics. Future research should focus on improving data quality and completeness, especially in rural and underrepresented areas, and integrating genetic, environmental, and lifestyle factors to provide a comprehensive understanding of stomach cancer risk. Comparative studies across different regions will be essential to identify successful intervention strategies and tailor them to specific demographic and regional needs. Additionally, distinguishing between cardia and non-cardia forms of stomach cancer in future analyses will help to elucidate their distinct etiologies and inform more precise prevention and treatment strategies. Enhanced screening programs and targeted interventions for high-risk populations are crucial for further reducing the incidence and mortality of stomach cancer in China.

## Supporting information

S1 Fig Distribution and rates of DALYs, YLDs, and YLLs due to stomach cancer in China, 2021. (A) Total DALYs by age and sex. (B) Age-standardized DALYs rate per 100,000 by age and sex. (C) Total YLDs by age and sex. (D) Age-standardized YLDs rate per 100,000 by age and sex. (E) Total YLLs by age and sex. (F) Age-standardized YLLs rate per 100,000 by age and sex. DALYs, disability-adjusted life years; YLDs, years lived with disability; YLLs, years of life lost.
(PDF)

S2 Fig Age, period, and cohort effects on the mortality of stomach cancer in China. (A) The age-specific mortality rates of stomach cancer according to time periods; each line connects the age-specific mortality rates for a 5-year period. (B) The period-specific mortality rates of stomach cancer according to age groups; each line connects the period-specific mortality rates for a 5-year age group. (C) The cohort-specific mortality rates of stomach cancer according to age groups; each line connects the cohort-specific mortality rates for a 5-year birth cohort. (D) The age-specific mortality rates of stomach cancer according to birth cohorts; each line connects the age-specific mortality rates for a 5-year birth cohort.
(PDF)

S3 Fig Decomposition analysis of changes in incidence and mortality of stomach cancer in China by aging, epidemiological change, and population growth. (A) Decomposition of changes in stomach cancer incidence for both sexes, males, and females. (B) Decomposition of changes in stomach cancer mortality for both sexes, males, and females.
(PDF)

S1 Table Change of age-standardized rates in incidence, prevalence, deaths, DALYs, YLDs, and YLLs for stomach cancer between 1990 and 2021 in China and global level. (DOCX)

S2 Table Joinpoint regression analysis of trends in age-standardized incidence, prevalence, mortality rates (per 100,000 persons) by sex from 1990 to 2021 for stomach cancer in China. (DOCX)

S3 Table Joinpoint regression analysis of trends in age-standardized DALYs, YLDs, and YLLs rates (per 100,000 persons) by sex from 1990 to 2021 for stomach cancer in China. (DOCX)
