# Supplementary figures and images for: Long-term trends and projections of stomach cancer burden in China: Insights from the GBD 2021 study
Source: PLoS One. 2025 Apr 8;20(4):e0320751. doi: 10.1371/journal.pone.0320751 (PMC11978042; doi:10.1371/journal.pone.0320751)

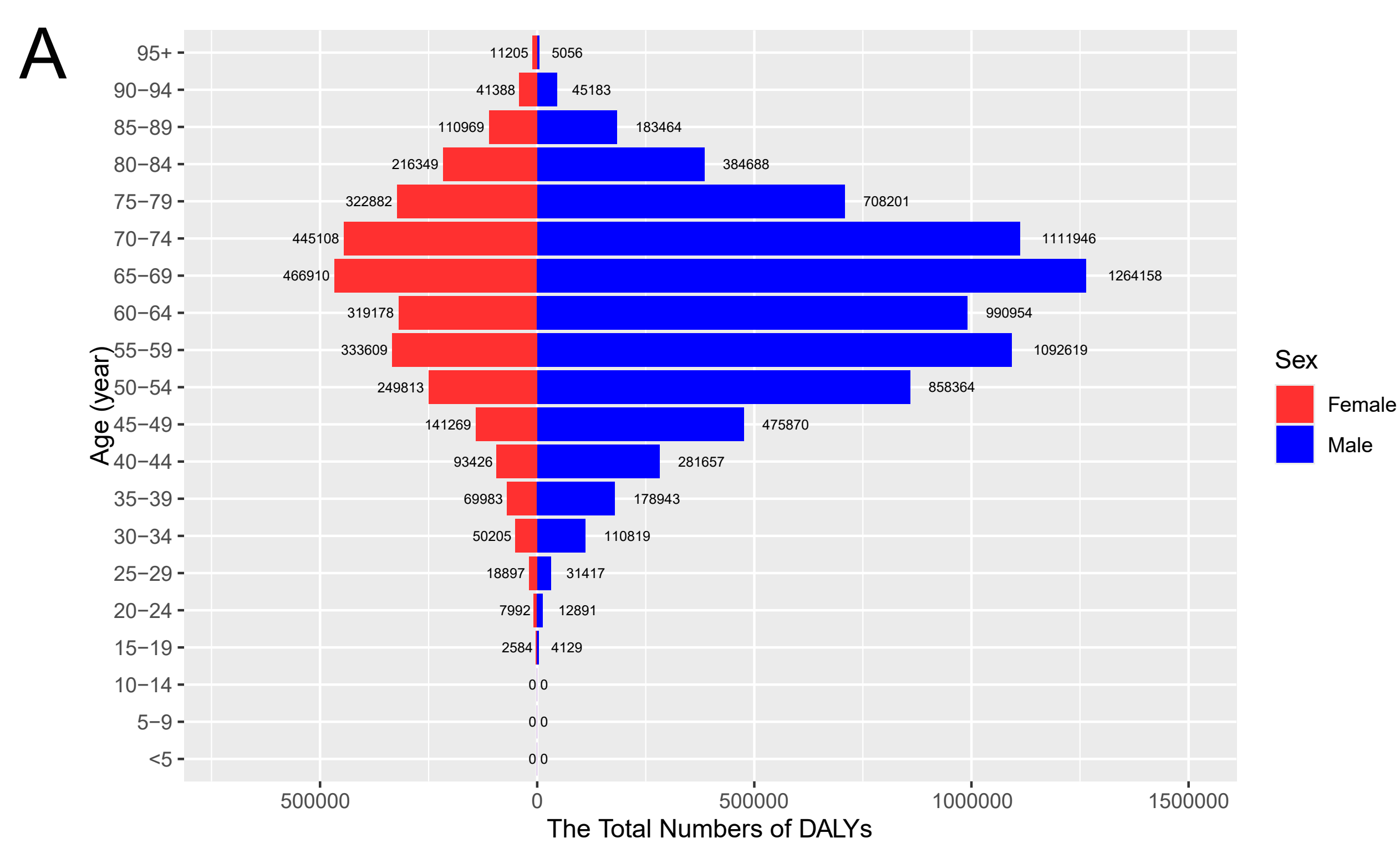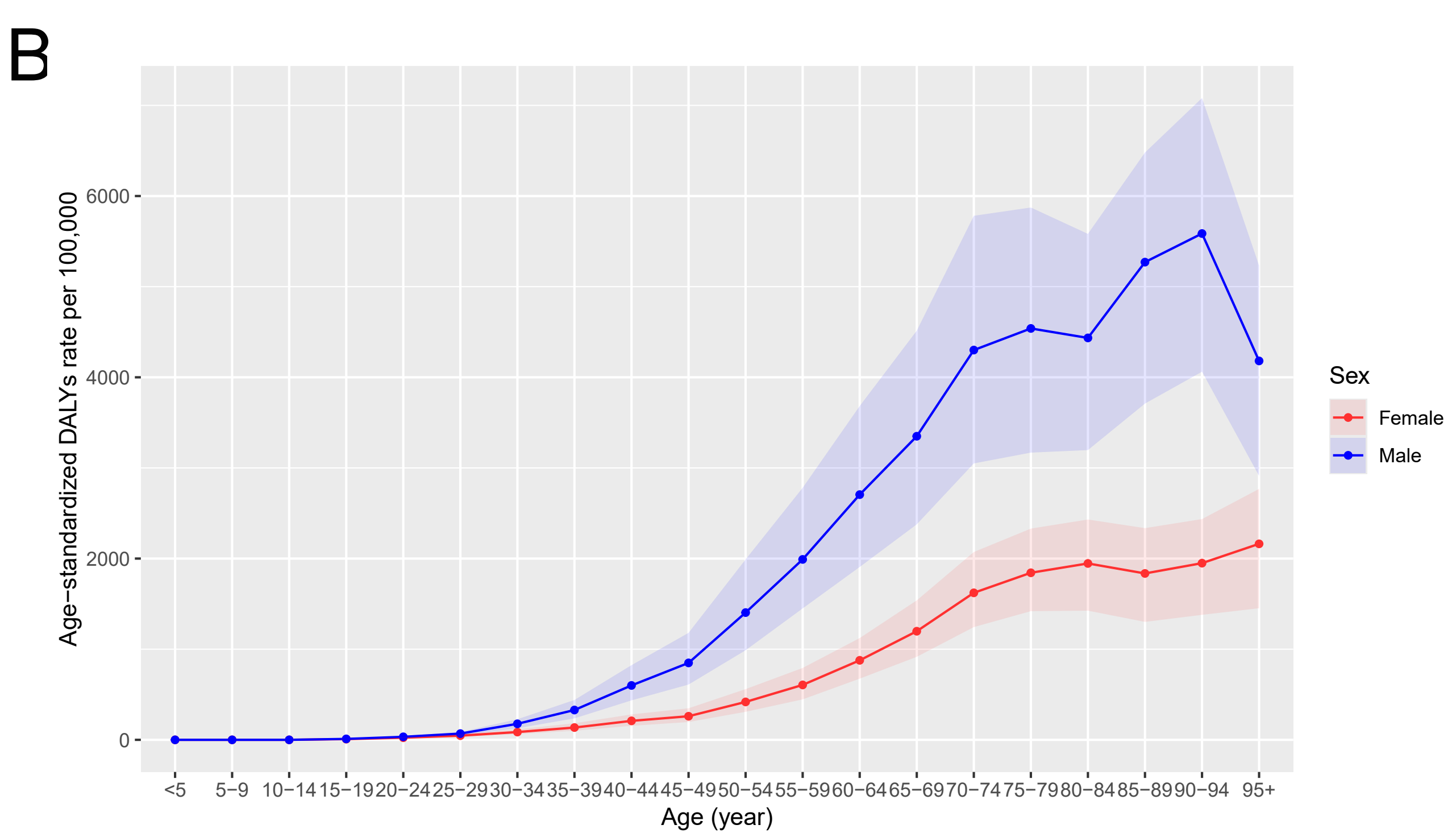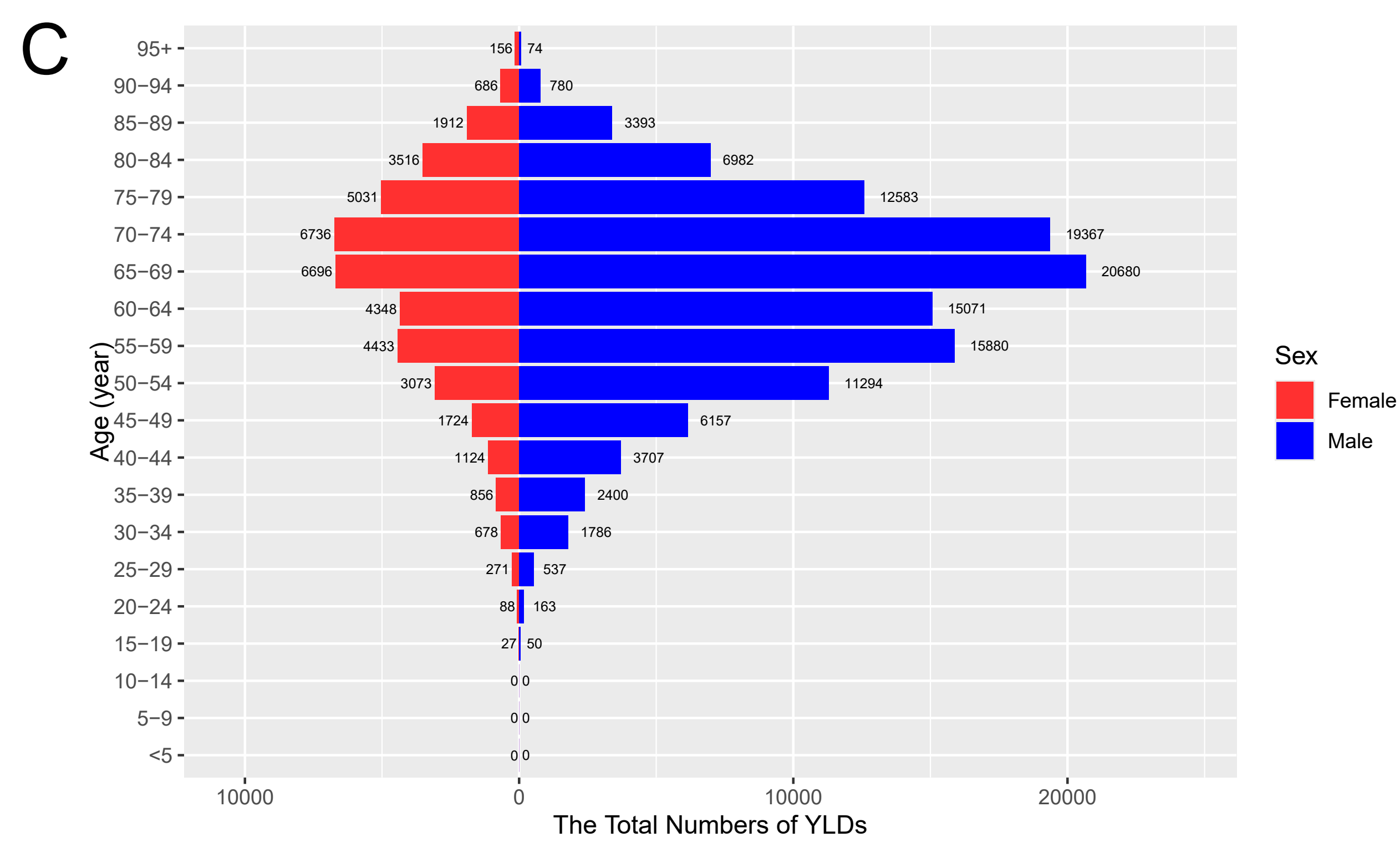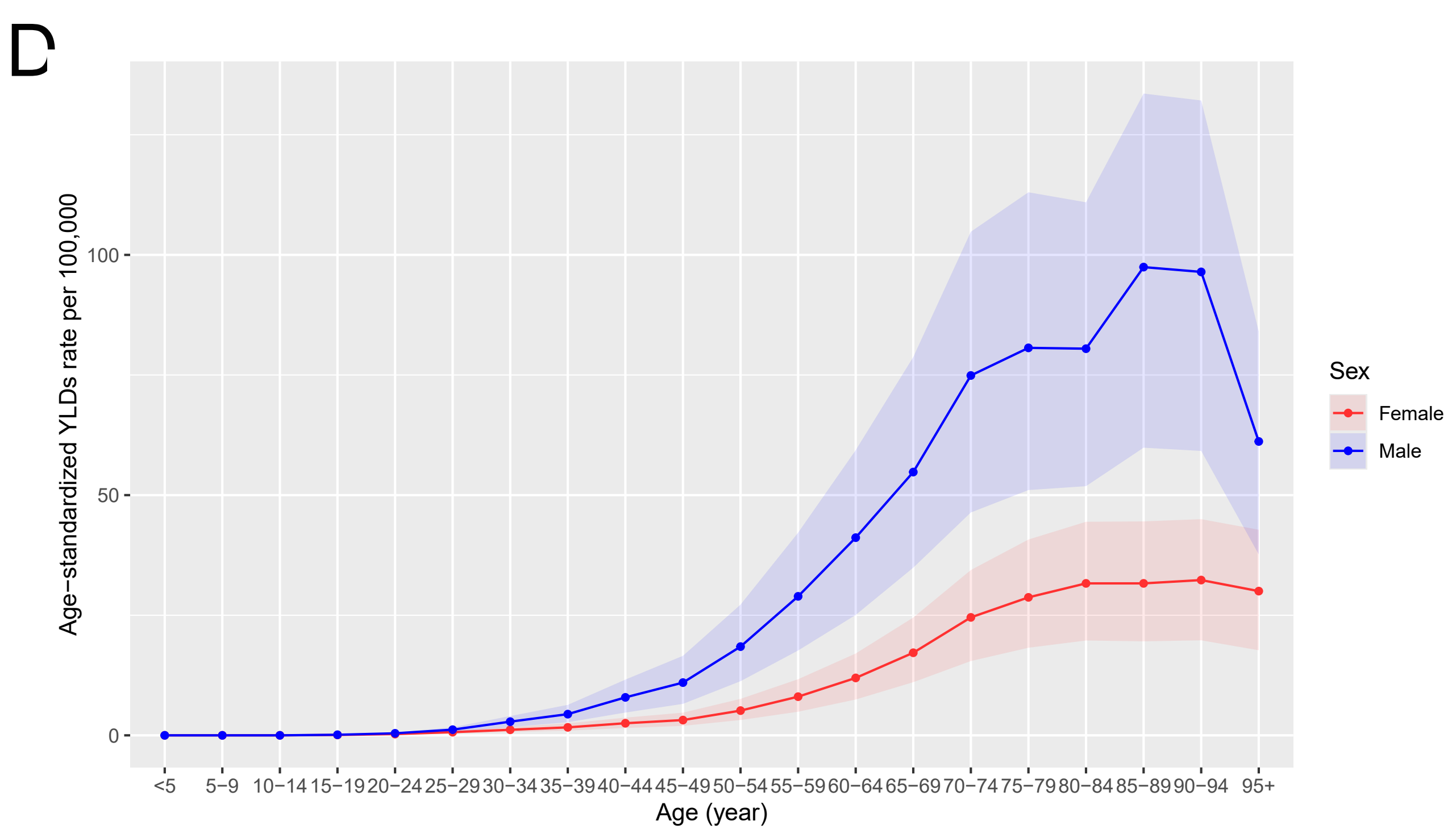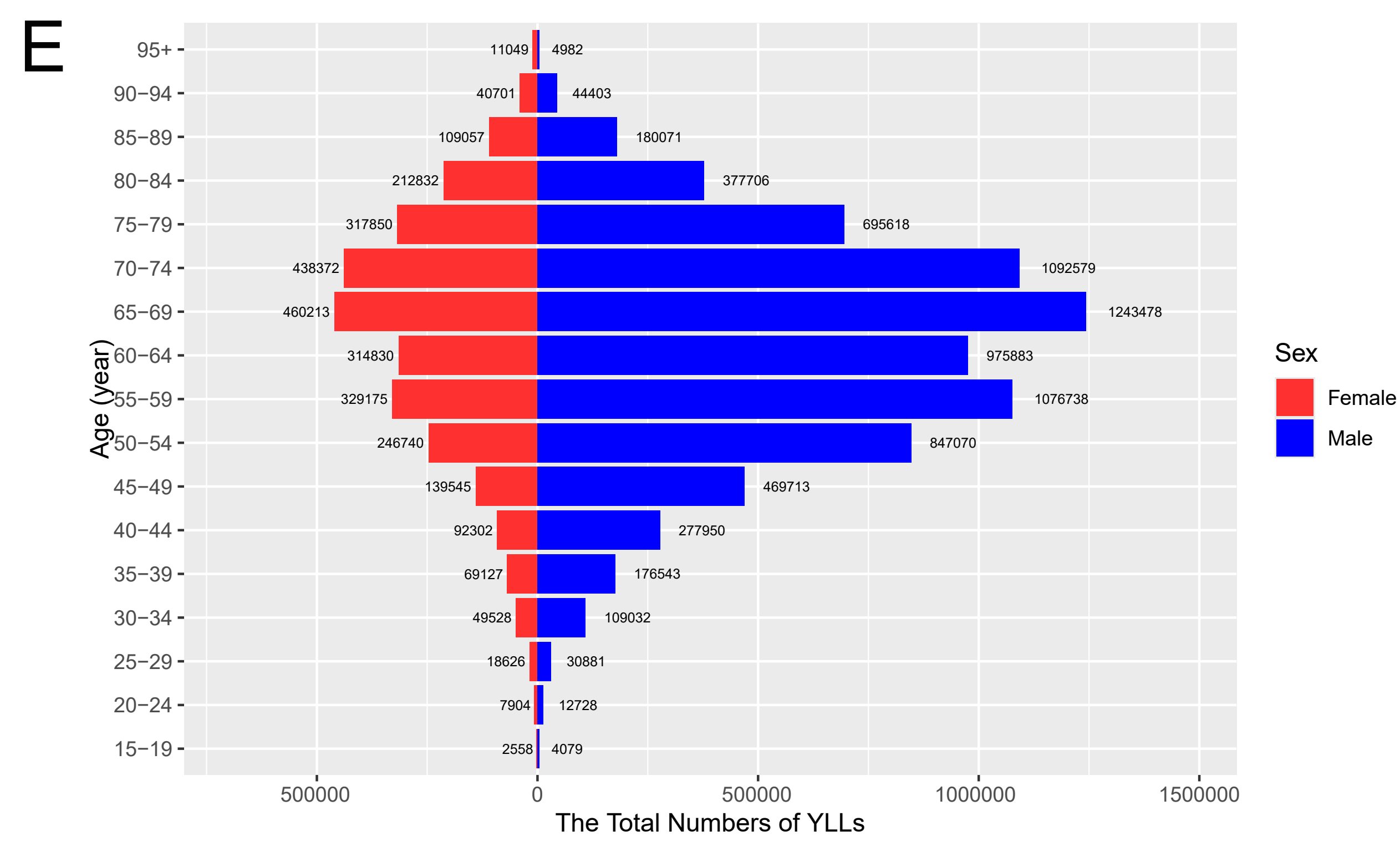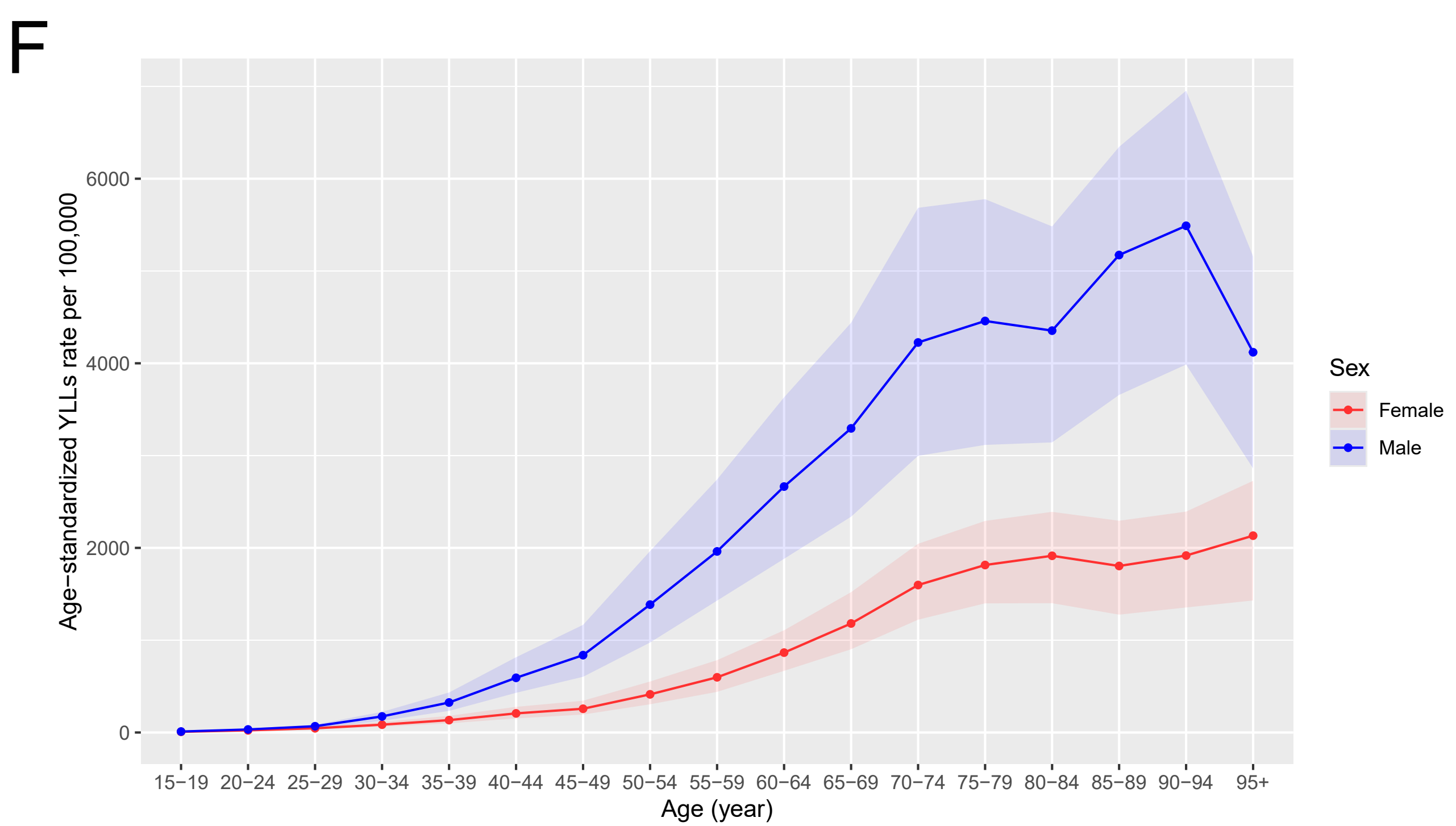

Supplement: S1 Fig — (A) Total DALYs by age and sex. (B) Age-standardized DALYs rate per 100,000 by age and sex. (C) Total YLDs by age and sex. (D) Age-standardized YLDs rate per 100,000 by age and sex. (E) Total YLLs by age and sex. (F) Age-standardized YLLs rate per 100,000 by age and sex. DALYs, disability-adjusted life years; YLDs, years lived with disability; YLLs, years of life lost. (PDF) [file pone.0320751.s001.pdf]

**A**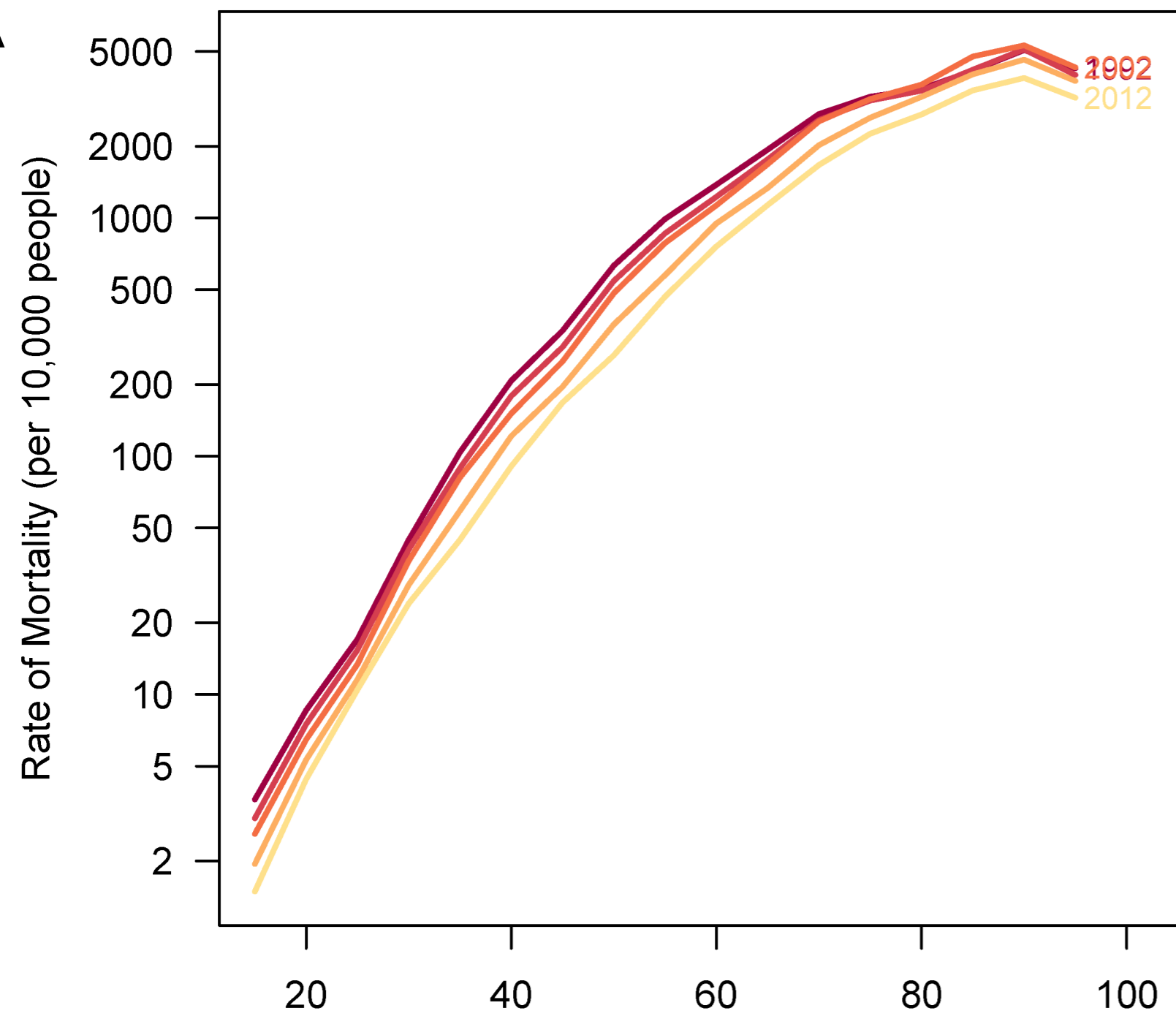**B**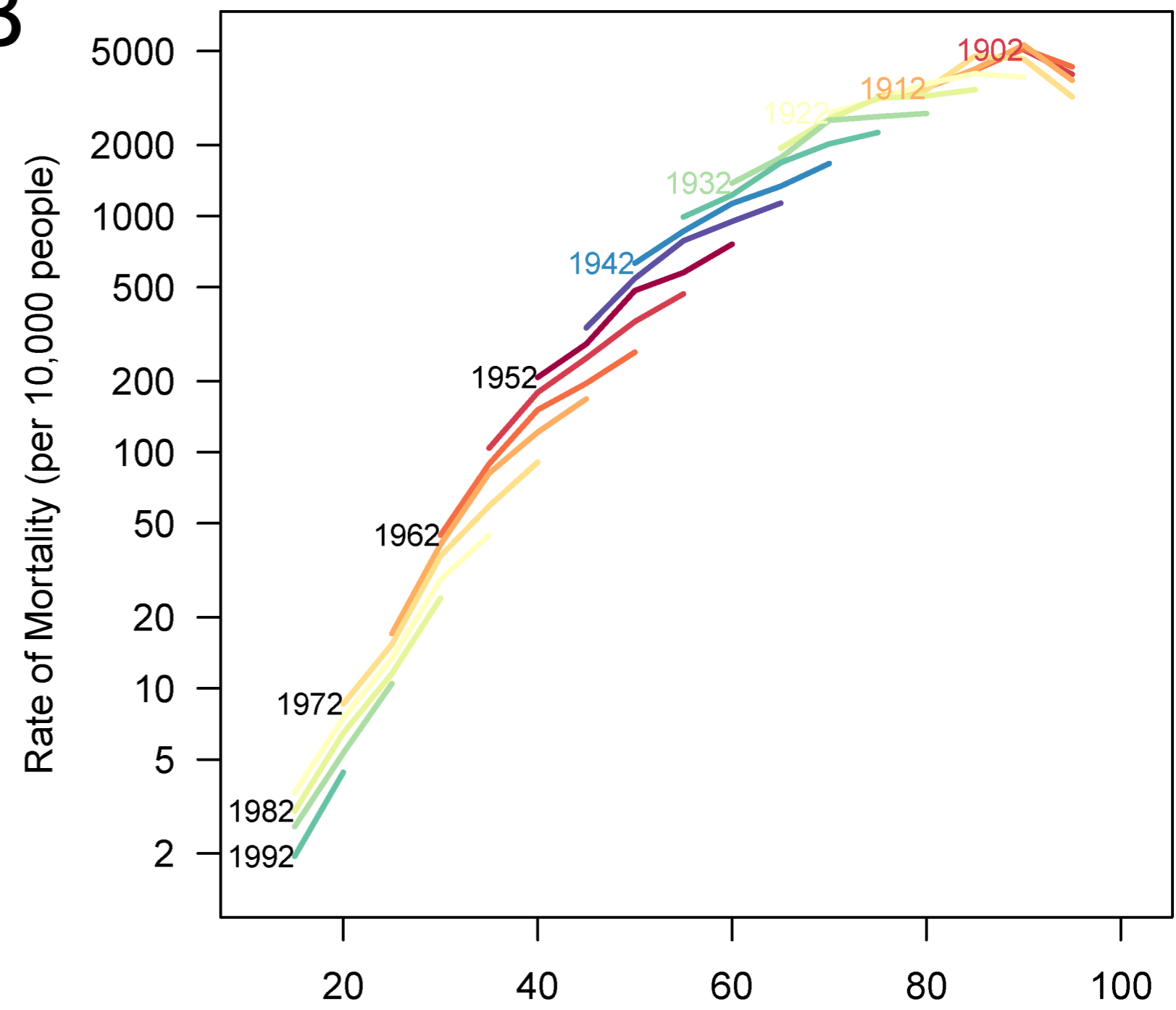**C**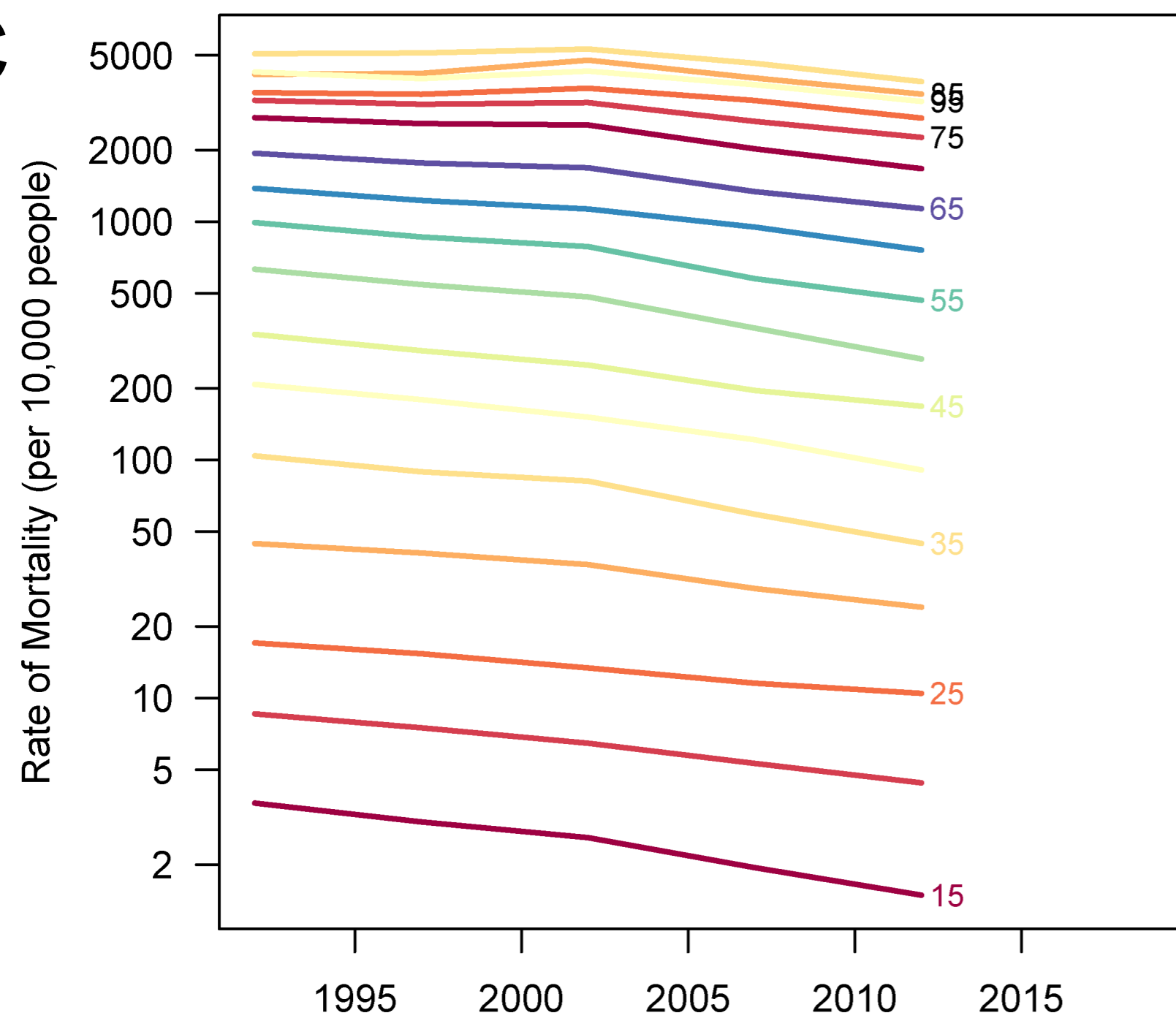**D**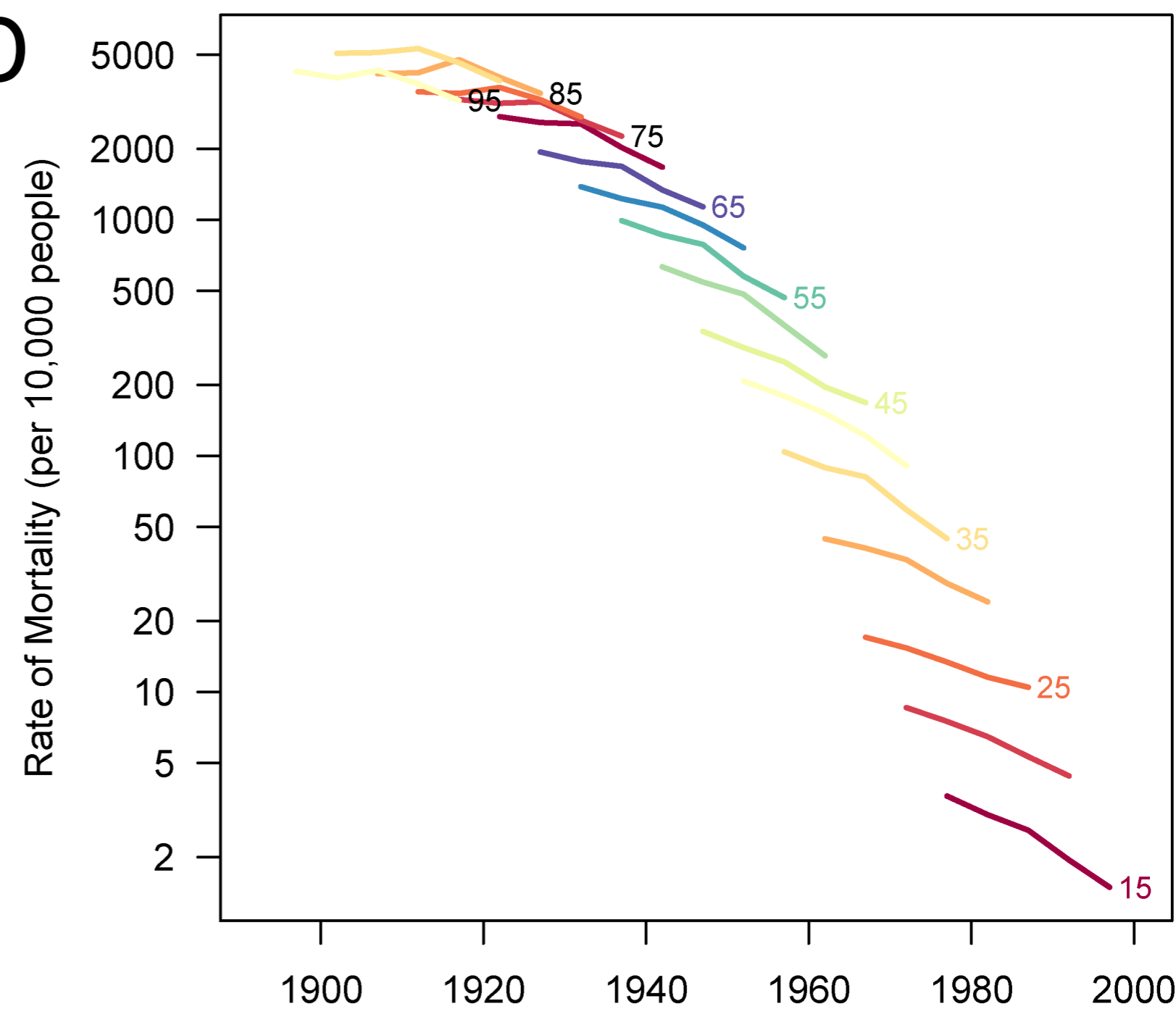

Supplement: S2 Fig — (A) The age-specific mortality rates of stomach cancer according to time periods; each line connects the age-specific mortality rates for a 5-year period. (B) The period-specific mortality rates of stomach cancer according to age groups; each line connects the period-specific mortality rates for a 5-year age group. (C) The cohort-specific mortality rates of stomach cancer according to age groups; each line connects the cohort-specific mortality rates for a 5-year birth cohort. (D) The age-specific mortality rates of stomach cancer according to birth cohorts; each line connects the age-specific mortality rates for a 5-year birth cohort. (PDF) [file pone.0320751.s002.pdf]

A

Incidence

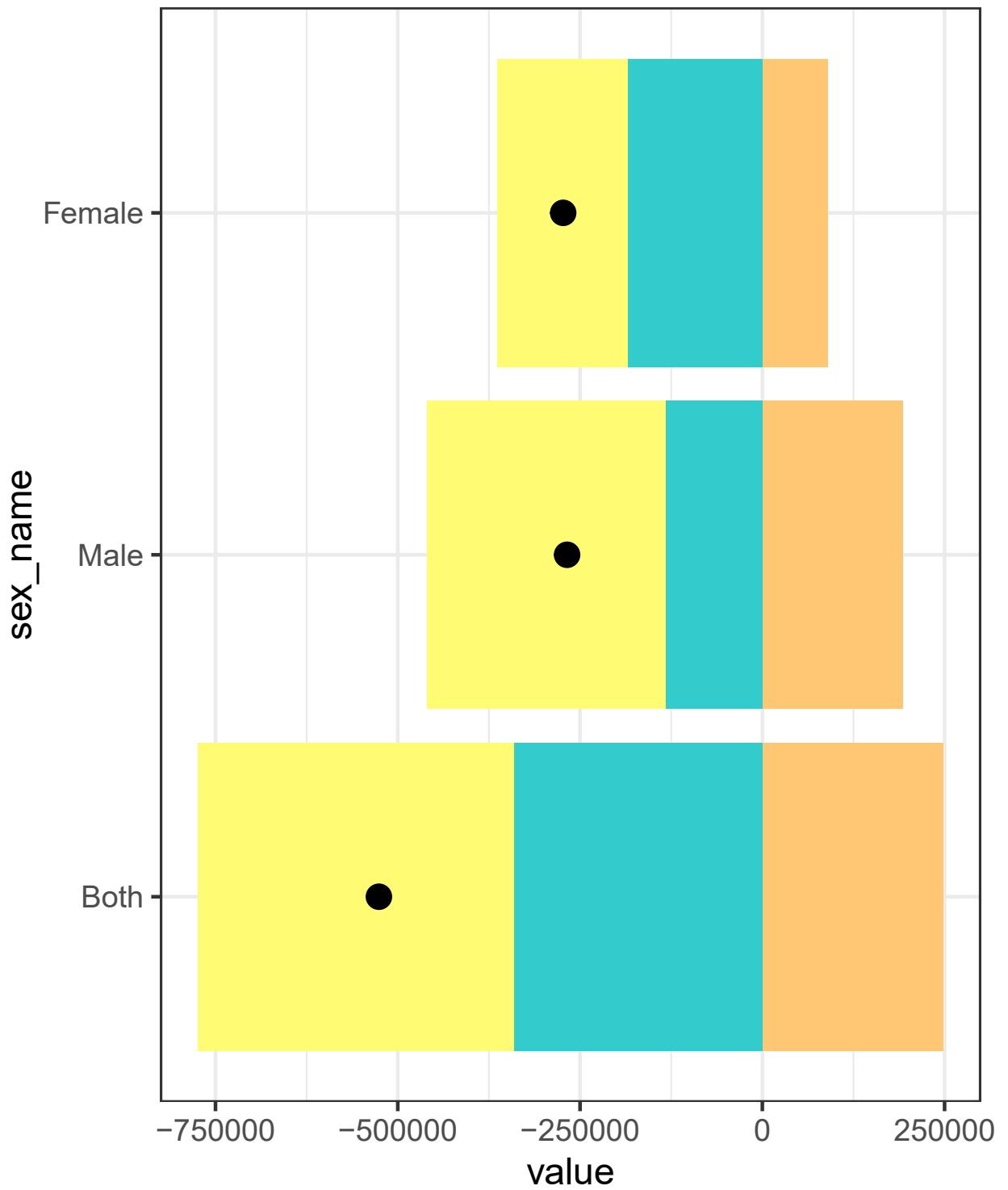

B

Mortality

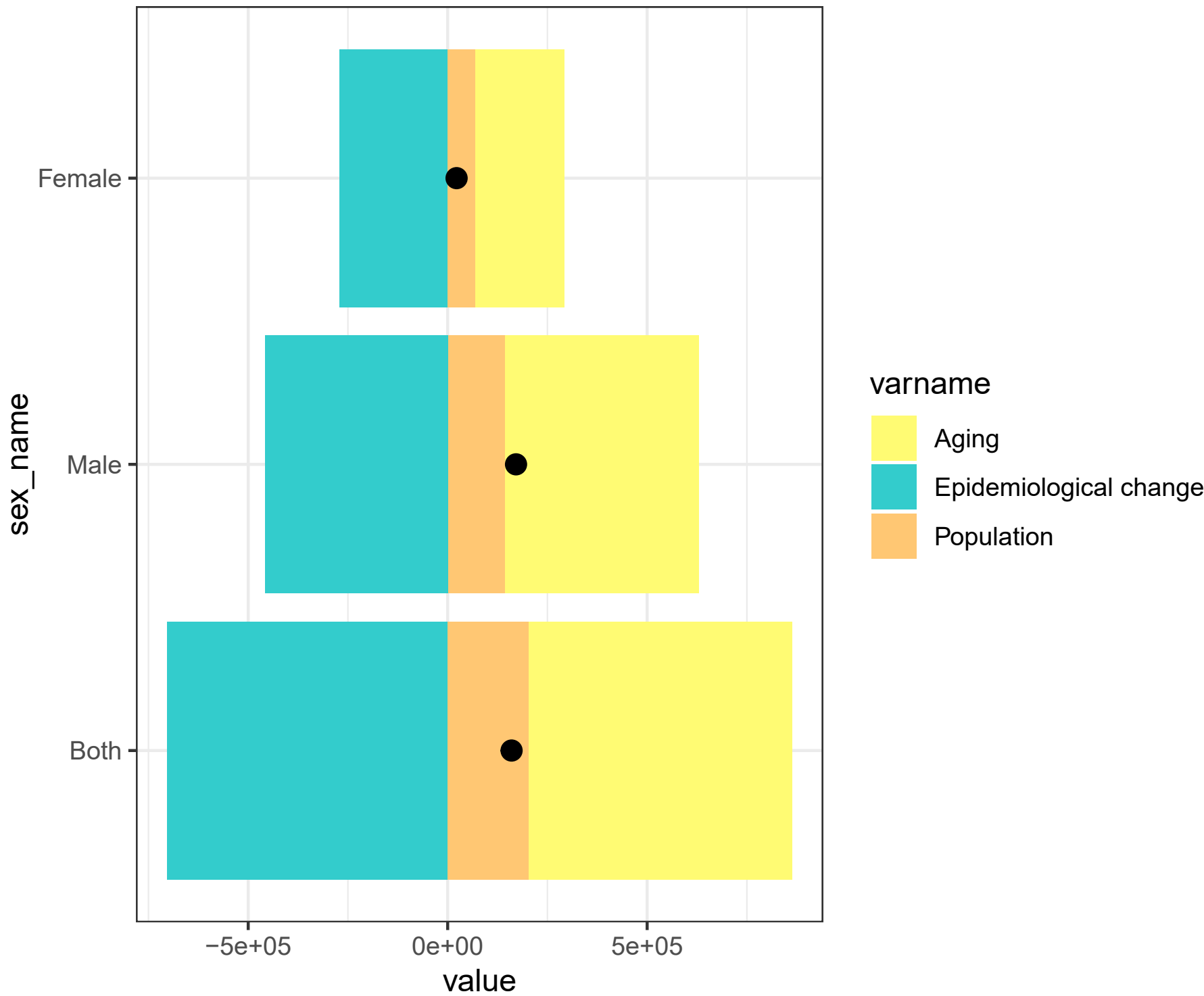

Supplement: S3 Fig — (A) Decomposition of changes in stomach cancer incidence for both sexes, males, and females. (B) Decomposition of changes in stomach cancer mortality for both sexes, males, and females. (PDF) [file pone.0320751.s003.pdf]
