# Supplementary material for: Long-term trends and projections of stomach cancer burden in China: Insights from the GBD 2021 study
Source: PLoS One. 2025 Apr 8;20(4):e0320751. doi: 10.1371/journal.pone.0320751 (PMC11978042; doi:10.1371/journal.pone.0320751)
Supplement: S1 Table — (DOCX) [file pone.0320751.s004.docx]

S1 Table. Change of age-standardized rates in incidence, prevalence, deaths, DALYs, YLDs, and YLLs for stomach cancer between 1990 and 2021 in China and global level.

| Measure | China | | | Global | | |
| --- | --- | --- | --- | --- | --- | --- |
|  | 1990 | 2021 | Change | 1990 | 2021 | Change |
| Incidence | 48.03 (40.21, 56.69) | 29.05 (22.42, 36.2) | -1.61 (-1.73 to -1.48) ^*^ | 24.76 (22.58, 27) | 14.33 (12.23, 16.41) | -1.76 (-1.89 to -1.64) ^*^ |
| Prevalence | 67.17 (55.35, 78.41) | 57.22 (44.18, 71.99) | -0.50 (-0.67 to -0.32) ^*^ | 40.64 (37.25, 43.68) | 27.58 (23.75, 31.89) | -1.26 (-1.40 to -1.12) ^*^ |
| Deaths | 46.05 (38.88, 54.43) | 21.51 (16.66, 26.61) | -2.34 (-2.60 to -2.07) ^*^ | 22.01 (20.03, 24.19) | 11.2 (9.62, 12.73) | -2.11 (-2.27 to -1.95) ^*^ |
| DALYs | 1181.61 (978.38, 1390.89) | 501.26 (387.29, 627.98) | -2.75 (-2.92 to -2.58) ^*^ | 559.72 (499.09, 615.77) | 262.75 (226.08, 301.02) | -2.42 (-2.52 to -2.33) ^*^ |
| LDs | 11.32 (7.68, 15.1) | 7.64 (5.13, 10.57) | -1.25 (-1.38 to -1.12) ^*^ | 6.14 (4.44, 8.01) | 3.77 (2.7, 4.92) | -1.57 (-1.70 to -1.44) ^*^ |
| YLLs | 1170.29 (969.14, 1379.01) | 493.62 (381.57, 620.17) | -2.65 (-2.86 to -2.43) ^*^ | 553.58 (493.58, 609.4) | 258.98 (223.19, 297.13) | -2.36 (-2.55 to -2.17) ^*^ |

DALYs, disability-adjusted life-years; YLDs, years lived with disability; YLLs, years of life lost; ^*^, *P*<0.05.
