# Supplementary material for: Long-term trends and projections of stomach cancer burden in China: Insights from the GBD 2021 study
Source: PLoS One. 2025 Apr 8;20(4):e0320751. doi: 10.1371/journal.pone.0320751 (PMC11978042; doi:10.1371/journal.pone.0320751)
Supplement: S2 Table — (DOCX) [file pone.0320751.s005.docx]

S2 Table. Joinpoint regression analysis of trends in age-standardized incidence, prevalence, mortality rates (per 100,000 persons) by sex from 1990 to 2021 for stomach cancer in China.

|  | ASIR |  |  | ASPR |  |  | ASMR |  |  |
| --- | --- | --- | --- | --- | --- | --- | --- | --- | --- |
| Gender | Period | APC (95% CI) | AAPC (95% CI) | Period | APC (95% CI) | AAPC (95% CI) | Period | APC (95% CI) | AAPC (95% CI) |
| Both | 1990-1998 | -1.84 (-1.93 to -1.75) ^*^ | -1.61 (-1.73 - -1.48) ^*^ | 1990-1998 | -1.02 (-1.14 to -0.89) ^*^ | -0.50 (-0.67 to -0.32) ^*^ | 1980-1998 | -2.10 (-2.18 to -2.03) ^*^ | -2.34 (-2.60 to -2.07) ^*^ |
|  | 1998-2004 | 0.96 (0.77 - 1.14) ^*^ |  | 1998-2004 | 2.30 (2.04 - 2.55) ^*^ |  | 1998-2004 | 0.30 (-0.18 - 0.78) |  |
|  | 2004-2007 | -4.38 (-5.18 to -3.57) ^*^ |  | 2004-2007 | -3.13 (-4.24 to -2.01) ^*^ |  | 2004-2007 | -6.13 (-8.23 to -3.97) ^*^ |  |
|  | 2007-2010 | -1.75 (-2.61 to -0.88) ^*^ |  | 2007-2010 | -0.74 (-1.94 - 0.47) |  | 2007-2010 | -2.75 (-5.03 to -0.41) ^*^ |  |
|  | 2010-2016 | -2.88 (-3.09 to -2.68) ^*^ |  | 2010-2015 | -1.74 (-2.14 to -1.34) ^*^ |  | 2010-2015 | -4.12 (-4.91 to -3.33) ^*^ |  |
|  | 2016-2021 | -0.97 (-1.23 to -0.72) ^*^ |  | 2015-2021 | -0.04 (-0.31 - 0.22) |  | 2015-2021 | -2.00 (-2.52 to -1.49) ^*^ |  |
| Female | 1990-1998 | -2.24 (-2.42 to -2.07) ^*^ | -2.17 (-2.33 - -2.02) ^*^ | 1990-1997 | -1.85 (-2.06 to -1.63) ^*^ | -1.29 (-1.44 to -1.14) ^*^ | 1980-1998 | -2.60 (-2.66 to -2.54) ^*^ | -2.82 (-2.98 to -2.67) ^*^ |
|  | 1998-2004 | -0.05 (-0.36 - 0.26) |  | 1997-2004 | 0.72 (0.50 - 0.93) ^*^ |  | 1998-2004 | -0.46 (-0.77 to -0.16) ^*^ |  |
|  | 2004-2007 | -5.47 (-6.70 to -4.21) ^*^ |  | 2004-2007 | -4.40 (-5.56 to -3.23) ^*^ |  | 2004-2007 | -7.19 (-8.43 to -5.94) ^*^ |  |
|  | 2007-2015 | -3.70 (-3.89 to -3.50) ^*^ |  | 2007-2015 | -2.78 (-2.96 to -2.60) ^*^ |  | 2007-2010 | -4.09 (-5.32 to -2.84) ^*^ |  |
|  | 2015-2021 | -0.44 (-0.80 to -0.08) ^*^ |  | 2015-2021 | 0.66 (0.30 - 1.02) ^*^ |  | 2010-2014 | -5.48 (-6.18 to -4.76) ^*^ |  |
|  |  |  |  |  |  |  | 2014-2021 | -1.39 (-1.66 to -1.11) ^*^ |  |
| Male | 1990-1998 | -1.70 (-1.83 to -1.56) ^*^ | -1.34 (-1.52 - -1.17) ^*^ | 1990-1998 | -0.82 (-0.98 to -0.67) ^*^ | -0.17 (-0.38 - 0.03) | 1980-1998 | -1.92 (-2.01 to -1.83) ^*^ | -2.11 (-2.41 to -1.81) ^*^ |
|  | 1998-2004 | 1.53 (1.26 - 1.80) ^*^ |  | 1998-2004 | 2.88 (2.57 - 3.20) ^*^ |  | 1998-2004 | 0.85 (0.26 - 1.43) ^*^ |  |
|  | 2004-2007 | -3.86 (-4.98 to -2.72) ^*^ |  | 2004-2007 | -2.58 (-3.90 to -1.24) ^*^ |  | 2004-2007 | -5.60 (-7.98 to -3.15) ^*^ |  |
|  | 2007-2010 | -1.10 (-2.32 - 0.14) |  | 2007-2010 | -0.09 (-1.51 - 1.35) |  | 2007-2010 | -2.13 (-4.73 - 0.55) |  |
|  | 2010-2017 | -2.57 (-2.79 to -2.35) ^*^ |  | 2010-2016 | -1.25 (-1.58 to -0.93) ^*^ |  | 2010-2015 | -3.84 (-4.69 to -2.99) ^*^ |  |
|  | 2017-2021 | -1.00 (-1.50 to -0.49) ^*^ |  | 2016-2021 | -0.00 (-0.40 - 0.39) |  | 2015-2021 | -2.36 (-2.91 to -1.79) ^*^ |  |

AAPC, average annual percent change presented for full period; APC, annual percent change; CI, confidence interval; ^*^, *P*<0.05.
