# Supplementary material for: Long-term trends and projections of stomach cancer burden in China: Insights from the GBD 2021 study
Source: PLoS One. 2025 Apr 8;20(4):e0320751. doi: 10.1371/journal.pone.0320751 (PMC11978042; doi:10.1371/journal.pone.0320751)
Supplement: S3 Table — (DOCX) [file pone.0320751.s006.docx]

S3 Table. Joinpoint regression analysis of trends in age-standardized DALYs, YLDs, and YLLs rates (per 100,000 persons) by sex from 1990 to 2021 for stomach cancer in China.

|  | DALYs |  |  | YLDs |  |  | YLLs |  |  |
| --- | --- | --- | --- | --- | --- | --- | --- | --- | --- |
| Gender | Period | APC (95% CI) | AAPC (95% CI) | Period | APC (95% CI) | AAPC (95% CI) | Period | APC (95% CI) | AAPC (95% CI) |
| Both | 1990-1998 | -2.57 (-2.69 to -2.44) ^*^ | -2.75 (-2.92 to -2.58) ^*^ | 1990-1998 | -1.61 (-1.71 to -1.51) ^*^ | -1.25 (-1.38 to -1.12) ^*^ | 1980-1998 | -2.37 (-2.46 to -2.29) ^*^ | -2.65 (-2.86 to -2.43) ^*^ |
|  | 1998-2004 | -0.35 (-0.60 to -0.10) ^*^ |  | 1998-2004 | 1.31 (1.11 - 1.50) ^*^ |  | 1998-2004 | -0.50 (-1.04 - 0.05) |  |
|  | 2004-2007 | -6.23 (-7.30 to -5.14) ^*^ |  | 2004-2007 | -3.91 (-4.77 to -3.05) ^*^ |  | 2004-2007 | -5.97 (-8.28 to -3.60) ^*^ |  |
|  | 2007-2010 | -3.40 (-4.57 to -2.22) ^*^ |  | 2007-2010 | -1.45 (-2.32 to -0.57) ^*^ |  | 2007-2015 | -3.95 (-4.30 to -3.59) ^*^ |  |
|  | 2010-2015 | -4.21 (-4.60 to -3.81) ^*^ |  | 2010-2016 | -2.41 (-2.61 to -2.21) ^*^ |  | 2015-2021 | -2.18 (-2.75 to -1.60) ^*^ |  |
|  | 2015-2021 | -2.04 (-2.31 to -1.77) ^*^ |  | 2016-2021 | -0.57 (-0.79 to -0.35) ^*^ |  |  |  |  |
| Female | 1990-1998 | -2.89 (-3.05 to -2.73) ^*^ | -3.25 (-3.38 - -3.11) ^*^ | 1990-1998 | -2.03 (-2.19 to -1.87) ^*^ | -1.89 (-2.05 - -1.73) ^*^ | 1980-1998 | -2.93 (-3.00 to -2.86) ^*^ | -3.19 (-3.32 to -3.05) ^*^ |
|  | 1998-2004 | -1.26 (-1.52 to -1.00) ^*^ |  | 1998-2004 | 0.15 (-0.17 - 0.47) |  | 1998-2004 | -1.25 (-1.59 to -0.90) ^*^ |  |
|  | 2004-2007 | -7.19 (-8.21 to -6.15) ^*^ |  | 2004-2007 | -5.08 (-6.41 to -3.73) ^*^ |  | 2004-2007 | -7.22 (-8.55 to -5.87) ^*^ |  |
|  | 2007-2014 | -5.20 (-5.41 to -5.00) ^*^ |  | 2007-2015 | -3.34 (-3.53 to -3.15) ^*^ |  | 2007-2014 | -5.22 (-5.49 to -4.95) ^*^ |  |
|  | 2014-2021 | -1.63 (-1.88 to -1.38) ^*^ |  | 2015-2021 | -0.14 (-0.42 - 0.15) |  | 2014-2021 | -1.64 (-1.97 to -1.32) ^*^ |  |
| Male | 1990-1998 | -2.45 (-2.61 to -2.28) ^*^ | -2.51 (-2.73 - -2.30) ^*^ | 1990-1998 | -1.47 (-1.60 to -1.33) ^*^ | -0.97 (-1.14 - -0.81) ^*^ | 1980-1998 | -2.17 (-2.27 to -2.07) ^*^ | -2.39 (-2.64 to -2.15) ^*^ |
|  | 1998-2004 | 0.16 (-0.17 - 0.49) |  | 1998-2004 | 1.88 (1.62 - 2.14) ^*^ |  | 1998-2004 | -0.04 (-0.66 - 0.58) |  |
|  | 2004-2007 | -5.73 (-7.11 to -4.33) ^*^ |  | 2004-2007 | -3.42 (-4.53 to -2.30) ^*^ |  | 2004-2007 | -5.39 (-7.95 to -2.76) ^*^ |  |
|  | 2007-2010 | -2.73 (-4.21 to -1.24) ^*^ |  | 2007-2010 | -0.81 (-1.96 - 0.35) |  | 2007-2017 | -3.44 (-3.71 to -3.17) ^*^ |  |
|  | 2010-2015 | -3.91 (-4.38 to -3.43) ^*^ |  | 2010-2017 | -2.07 (-2.27 to -1.86) ^*^ |  | 2017-2021 | -1.95 (-3.12 to -0.76) ^*^ |  |
|  | 2015-2021 | -2.32 (-2.64 to -2.01) ^*^ |  | 2017-2021 | -0.55 (-0.98 to -0.13) ^*^ |  |  |  |  |

DALYs, disability-adjusted life years; YLDs, years lived with disability; YLLs, years of life lost; AAPC, average annual percent change presented for full period; APC, annual percent change; CI, confidence interval. ^*^, *P*<0.05.
